# Prediction of cardiovascular events in rheumatoid arthritis using risk age calculations: evaluation of concordance across risk age models

**DOI:** 10.1186/s13075-020-02178-z

**Published:** 2020-04-23

**Authors:** Grunde Wibetoe, Joseph Sexton, Eirik Ikdahl, Silvia Rollefstad, George D. Kitas, Piet van Riel, Sherine Gabriel, Tore K. Kvien, Karen Douglas, Aamer Sandoo, Elke E. Arts, Solveig Wållberg-Jonsson, Solbritt Rantapää Dahlqvist, George Karpouzas, Patrick H. Dessein, Linda Tsang, Hani El-Gabalawy, Carol A. Hitchon, Virginia Pascual-Ramos, Irazu Contreas-Yañes, Petros P. Sfikakis, Miguel A. González-Gay, Iris J. Colunga-Pedraz, Dionicio A. Galarza-Delgado, Jose Ramon Azpiri-Lopez, Cynthia S. Crowson, Anne Grete Semb

**Affiliations:** 1 Preventive Cardio-Rheuma clinic, Department of Rheumatology, Diakonhjemmet Hospital, PO Box 23, Vindern, N-01319 Oslo, Norway; 2 Department of Rheumatology, Diakonhjemmet Hospital, Oslo, Norway; 3 School of Sport, Exercise and Rehabilitation, University of Birmingham, Birmingham, UK; 4 Radboud Institute for Health Sciences, IQ healthcare, Radboud University Medical Center, Nijmegen, The Netherlands; 5 Rutgers Robert Wood Johnson Medical School, New Brunswick, NJ USA; 6 Dudley Group NHS Foundation Trust, West Midlands, UK; 7 School of Sport, Health and Exercise Sciences, Bangor University, Bangor, UK; 8 Department of Rheumatic Diseases, Radboud University Nijmegen Medical Centre, Nijmegen, Netherlands; 9 Department of Public Health and Clinical Medicine, Rheumatology, Umeå University, Umeå, Sweden; 10 Division of Rheumatology, Harbor-UCLA Medical Center, Torrance, CA USA; 11 Vrije Universiteit Brussel, Brussels, Belgium; 12 Universitair Ziekenhuis Brussel, Brussels, Belgium; 13 Rheumatology, Universitair Ziekenhuis Brussel, Brussels, Belgium; 14 University of Manitoba, Winnipeg, MB Canada; 15 Instituto Nactional de Ciencias Médicas y Nutrición Salvador Zubirán, México City, Mexico; 16 First Department of Propedeutic Internal Medicine, National and Kapodistrian University of Athens, Athens, Greece; 17 Rheumatology, Hospital Universitario Marqués de Valdecilla, IDIVAL, Universidad de Cantabria, Santander, Spain; 18 Rheumatology, Hospital Universitario, UANL, Monterrey, Mexico; 19 Hospital Universitario, UANL, Monterrey, Mexico; 20 Cardiology, Hospital Universitario, UANL, Monterrey, Mexico; 21 Division of Rheumatology, Department of Medicine, Mayo Clinic, Rochester, MN USA

**Keywords:** Cardiovascular risk age, Vascular age, Cardiovascular disease, Risk factors, Rheumatoid arthritis

## Abstract

**Background:**

In younger individuals, low absolute risk of cardiovascular disease (CVD) may conceal an increased risk age and relative risk of CVD. Calculation of risk age is proposed as an adjuvant to absolute CVD risk estimation in European guidelines. We aimed to compare the discriminative ability of available risk age models in prediction of CVD in rheumatoid arthritis (RA). Secondly, we also evaluated the performance of risk age models in subgroups based on RA disease characteristics.

**Methods:**

RA patients aged 30–70 years were included from an international consortium named A Trans-Atlantic Cardiovascular Consortium for Rheumatoid Arthritis (ATACC-RA). Prior CVD and diabetes mellitus were exclusion criteria. The discriminatory ability of specific risk age models was evaluated using c-statistics and their standard errors after calculating time until fatal or non-fatal CVD or last follow-up.

**Results:**

A total of 1974 patients were included in the main analyses, and 144 events were observed during follow-up, the median follow-up being 5.0 years. The risk age models gave highly correlated results, demonstrating *R*^2^ values ranging from 0.87 to 0.97. However, risk age estimations differed > 5 years in 15–32% of patients. C-statistics ranged 0.68–0.72 with standard errors of approximately 0.03. Despite certain RA characteristics being associated with low c-indices, standard errors were high. Restricting analysis to European RA patients yielded similar results.

**Conclusions:**

The cardiovascular risk age and vascular age models have comparable performance in predicting CVD in RA patients. The influence of RA disease characteristics on the predictive ability of these prediction models remains inconclusive.

## Background

Patients with rheumatoid arthritis (RA) have higher risk of cardiovascular disease (CVD) [1]. Risk algorithms for the general population lack precision when applied to RA patients, while validated RA-specific CVD prediction models are currently lacking [2–5].

Numerous CVD risk prediction models have been developed and differ in terms of CVD events (fatal or non-fatal), patient population of interest, and also CVD risk factors (CVD-RFs) included [6]. In clinical practice, the decision whether to initiate lipid-lowering therapy or not is supported by calculating the absolute risk of CVD [7]. Using the Systematic Coronary Risk Evaluation (SCORE) algorithm, the absolute 10-year risk of a fatal CVD event can be estimated separately for patients in countries with low and high risk of CVD [8]. The SCORE algorithm estimates CVD risk according to age, sex, and modifiable CVD-RFs including smoking status, systolic blood pressure (sBP), and total cholesterol (TC). Since high-density lipoprotein cholesterol (HDL-c) may improve prediction of CVD, an updated algorithm includes TC to HDL-c ratio [9, 10]. The European League Against Rheumatism (EULAR) advises to adapt CVD risk prediction models with a 1.5 multiplication factor to the estimated risk of CVD for patients with RA [11].

Absolute risk of CVD the next 10 years is largely driven by age, and younger individuals will thus have a low calculated risk by SCORE, even in the presence of high levels of CVD-RFs [12]. Accordingly, a low absolute 10-year risk may conceal a high relative and lifetime risk of CVD [7]. Risk age may be a favorable supplement for assessment and communication of CVD risk, and its use is recommended in the guidelines on CVD prevention by the European Society of Cardiology (ESC) in addition to assessment of absolute 10-year risk [7].

The risk age concept estimates the detrimental effects of CVD-RFs, by comparing the absolute risk of the patient of interest to the absolute risk in a hypothetical individual of the same sex with the absence of CVD-RFs (a non-smoker with sBP of 120 mmHg and TC at 4 mmol/L). Estimated risk age for any given individual may be similar to his/her true age or far beyond the chronologic age in presence of high levels of CVD-RFs. If a 40-year-old has a risk age of 60 years, then he/she has an absolute risk corresponding to a 60-year-old person of the same sex without CVD-RFs (non-smoker with sBP of 120 mmHg and TC of 4 mmol/L). Currently, two risk age models based on the SCORE algorithm have been developed: the cardiovascular risk age and the vascular age [13, 14].

Recently, we reported that different CVD risk age models yield highly correlated results, yet differ by 5 years or more in a substantial fraction of RA patients, suggesting a need for validation of these prediction models [15]. Our objective was to compare the discriminative ability of cardiovascular risk age and vascular age calculations in predicting CVD among RA patients. We also aimed to evaluate the performance of these risk age models in subgroups of RA patients stratified according to rheumatic disease related characteristics.

## Methods

### Study populations

Patients were included from the international consortium, A Trans-Atlantic Cardiovascular Consortium for Rheumatoid Arthritis (ATACC-RA), which encompasses 11 RA patient cohorts (the UK, Norway, Netherlands, Sweden, Greece, Spain, the USA, South Africa, Canada, Mexico (2 cohorts)) [3]. Approval was granted by the ethical boards/committees at each center. Data were anonymized and aggregated before analyses.

In the current analyses, we included patients aged 30–70 years at baseline. Exclusion criteria were prior CVD and/or diabetes mellitus at baseline in line with European guidelines (as CVD risk prediction is limited to patients without known prior CVD and/or diabetes mellitus). For main analyses, we also excluded patients using lipid-lowering therapy (LLT) and/or antihypertensive treatment (AntiHT) at baseline. In secondary analyses, we performed analyses on data from European RA cohorts (subanalyses 1), included RA patients on LLT and/or AntiHT (subanalyses 2), and performed analyses on European RA cohorts also including patients on LLT and/or AntiHT (subanalyses 3).

### Demographic and rheumatic disease characteristics

Age, sex, and RA disease-related variables (disease duration, disease activity score using 28 joint count (DAS28), rheumatoid factor (RF) and anti-citrullinated protein antibodies (ACPA), and treatment with glucocorticoids (GC), synthetic and biologic disease-modifying anti-rheumatic drugs (bDMARDs and sDMARDs)) were collected at baseline. Recorded CVD-RFs included current smoking, sBP, TC, and HDL-c; body mass index (BMI), low-density lipoprotein cholesterol (LDL-c), and triglycerides were also recorded.

### Outcomes

The outcome of interest was time to physician-verified fatal or non-fatal CVD events, including CVD death, myocardial infarction, stroke, peripheral arterial disease (with or without revascularization procedures), and coronary revascularization (percutaneous coronary intervention or coronary artery bypass grafting). Patients were followed from baseline to the time of event or censored at the time of last follow-up.

### Relative risk calculation

Relative risk of CVD was calculated according to the relative risk chart, which is included in the 2016 European guidelines on CVD prevention in clinical practice, in accordance with smoking status as well as total cholesterol and systolic blood pressure levels [7].

### Risk age estimations using the cardiovascular risk age model

Cardiovascular risk age was estimated according to the cardiovascular risk age chart, based on the nearest intervals of sBP (120/140/160/180 mmHg) and TC (4/5/6/7/8 mmol/L) for males and females, depending on different chronologic age strata (40/45/50/55/60/65 years) [13].

### Risk age estimations using the vascular age model

In contrast, the vascular age calculation was performed in two steps. First, we estimated 10-year risk of fatal CVD according to the SCORE algorithms [8, 10]. Then, we matched calculated 10-year risk to corresponding risk ages [14]. The vascular age was calculated in four different ways, corresponding to the four different SCORE formulas for assessing the 10-year risk of fatal CVD: using the formula for low-risk countries and the formula high-risk countries, as well as those with and without HDL-c.

### Statistical analysis

Descriptive data was presented as counts (percentages) for categorical data and as mean or median for normally distributed and non-normally distributed data, respectively. The significance level was set at *p* < 0.05.

Since the cardiovascular risk age model was developed for patients aged 40–65, ages 30–40 and 65–70 years were truncated to 40 and 65 years, respectively. Agreement between risk age models was evaluated using *R*^2^ from a linear regression model and the frequency of risk age estimations differing 5 years or more across models.

To validate the CVD risk prediction models, discrimination (e.g., correctly ranking patients who experience CVD as individuals at higher risk than those who do not experience CVD) was calculated using concordance statistics (c-statistics) [16, 17]. Moreover, c-statistics with standard errors were compared across risk age models to evaluate which risk age models perform better at ranking individuals correctly as low- or high-risk individuals (in which the latter should have shorter observed time to event). Concordance was also estimated for individual centers.

Furthermore, concordance according to sex and baseline RA disease characteristics was calculated to assess whether these features influence the risk age model’s predictive ability. In detail, these RA disease characteristics included (1) disease duration ≤ 1 vs. > 1 year, (2) disease activity (remission/low disease activity vs. moderate/high disease activity according to DAS28) [18, 19], (3) seropositivity (RF and/or ACPA positivity), and presence or absence of anti-rheumatic treatment with (4) glucocorticoids, (5) methotrexate, (6) sDMARDs in general, or (7) bDMARDs. The sampling variability of these sub-group c-statistics was assessed using standard errors.

Statistical analyses were conducted using R 3.2.0 (R Foundation for Statistical Computing, Vienna, Austria) and STATA version 14.1.

## Results

Table 1 presents baseline characteristics of the 1973 RA patients included in the main analyses.

**Table 1:** Patient characteristics

	Main population (***n*** = 1974)	Sub-analyses 1 (***n*** = 1543)	Sub-analyses 2 (***n*** = 2617)	Sub-analyses 3 (***n*** = 1991)
Follow-up time in years, median (IQR)	5 (2.6,9.1)	5 (2.7,10.3)	5 (2.3,7.7)	5 (2.5,8.5)
Age in years, median (IQR)	52.0 (43.7,59.4)	53.1 (45,60.5)	54 (45.5,61)	55.3 (46.8,62)
Females, *n* (%)	1465 (74%)	1095 (71%)	1955 (75%)	1432 (72%)
Disease duration in years, median (IQR)	0.6 (0.1,5.8)	0.4 (0,4.3)	0.8 (0.1,7.6)	0.6 (0.1,6)
Rheumatoid factor, *n* (%)	1437 (73%)	1078 (70%)	1907 (74%)	1392 (70%)
Anti-cyclic citrullinated peptide antibody, *n* (%)	1239 (66%)	905 (62%)	1649 (67%)	1177 (63%)
Glucocorticosteroids, *n* (%)	469 (24%)	345 (22%)	675 (26%)	502 (25%)
Biologic DMARD, *n* (%)	193 (10%)	149 (10%)	321 (12%)	239 (12%)
Synthetic DMARD, *n* (%)	722 (38%)	517 (34%)	1056 (43%)	743 (37%)
Methotrexate, *n* (%)	581 (30%)	365 (24%)	864 (34%)	529 (27%)
DAS 28, mean (SD)	4.2 (1.6)	4.3 (1.5)	4.1 (1.6)	4.2 (1.5)
Remission (< 2.6)	334 (18%)	206 (14%)	491 (20%)	280 (15%)
Low disease activity (2.6, 3.2)	169 (9%)	141 (10%)	234 (9%)	187 (10%)
Moderate disease activity (3.2, 5.1)	810 (43%)	681 (47%)	1051 (42%)	876 (47%)
High disease activity (> 5.1)	566 (30%)	433 (30%)	704 (28%)	540 (29%)
CRP (mg/l), median (IQR)	6.2 (1.4, 18)	8 (2, 21)	6.2 (1.4, 17)	8 (2, 20)
ESR (mm/h), median (IQR)	20 (10, 35.9)	20 (10, 36)	20 (10, 36)	20 (10, 36)
TJC 28, median (IQR)	4 (1, 8)	4 (2, 8)	4 (1, 8)	4 (1, 8)
SJC 28, median (IQR)	5 (2, 9)	5 (2, 9)	4 (1, 9)	5 (2, 9)
Total cholesterol (mmol/l), median (IQR)	5.2 (4.4, 5.9)	5.3 (4.5, 6.1)	5.2 (4.4, 6)	5.4 (4.6, 6.1)
HDL-c (mmol/l), median (IQR)	1.4 (1.1, 1.7)	1.4 (1.2, 1.7)	1.4 (1.1, 1.7)	1.4 (1.2, 1.7)
LDL-c (mmol/l), median (IQR)	3.1 (2.4, 3.8)	3.2 (2.6, 4)	3.1 (2.4, 3.8)	3.2 (2.6, 3.9)
Triglyceride (mmol/l), median (IQR)	1.2 (0.9, 1.7)	1.2 (0.9, 1.7)	1.2 (0.9, 1.7)	1.2 (0.9, 1.7)
Systolic BP (mm Hg), mean (SD)	134.1 (21.4)	137.7 (21.2)	136.1 (21.8)	139.7 (21.7)
Diastolic BP (mm Hg), mean (SD)	80.1 (10.8)	81.4 (10.5)	80.8 (11)	82.2 (10.7)
BMI (kg/m^2^), median (IQR)	25.6 (23.0, 28.7)	25.4 (22.9, 28.4)	26.1 (23.4, 29.5)	25.9 (23.3, 29.3)
BMI ≥ 30 kg/m^2^, *n* (%)	324 (19%)	226 (17%)	523 (23%)	366 (22%)
Current smokers, *n* (%)	560 (30%)	497 (34%)	678 (28%)	599 (32%)

*IQR* inter-quartile range, *SD* standard deviation, *bDMARDs and sDMARDs* biologic and synthetic disease-modifying antirheumatic drugs, *DAS28* disease activity score using 28 joint count, *CRP* C-reactive protein, *ESR* erythrocyte sedimentation rate, *HDL-c* high-density lipoprotein cholesterol, *sBP and dBP* systolic and diastolic blood pressure. Baseline characteristics describing demographic data, rheumatoid arthritis-related disease characteristics, and cardiovascular risk factors in rheumatoid arthritis at baseline. Data are also specified on patients who do and do not experience CVD events during follow-up. In subanalyses 1, only European rheumatoid arthritis cohorts were included. In subanalyses 2, rheumatoid arthritis patients on lipid-lowering therapy and/or antihypertensive treatment were included. In subanalyses 3, analyses were performed on patients from European rheumatoid arthritis cohorts also including patients on lipid-lowering therapy and/or antihypertensive treatment

Seventy-four percent were female, and median (inter-quartile range) age was 52.0 (44, 59) years. Disease duration was 0.6 (0.1, 5.8) years, and more than half (61%) had an RA disease duration of 1 year or less. Overall, 73% were RF positive. There were a substantial number of patients with moderate (43%) or high (30%) disease activity according to DAS28. Twenty-four percent were treated with glucocorticoids, while 10% and 38% were using bDMARDs and sDMARDs, respectively. About a third (30%) were current smokers, and 19% were obese (BMI ≥ 30kg/m^2^).

The median follow-up time was 5.0 (2.5, 9.1) years. A total of 144 RA patients experienced CVD events during the observation period: confirmed myocardial infarction (*n* = 64), stroke (*n* = 33), CVD death (*n* = 19), peripheral arterial events (*n* = 14), and revascularization procedures (*n* = 14). Among the patients included in the main analyses, estimation of risk age was not possible in 338 (17%) and 357 (18%) individuals when using prediction models without or with HDL-c, respectively, due to missing data on sBP (*n* = 114), TC (*n* = 205), HDL-c (*n* = 219), and current smoking (*n* = 119).

The estimated risk of CVD according to different prediction models are presented in Table 2. Calculated risk age was highest when the cardiovascular risk age model was applied and, depending on the specific risk age model used, the median risk age ranged from 56 to 59 years. According to the relative risk table, two thirds of patients with RA had at least twice the risk of CVD (due to either smoking and/or elevated sBP/TC levels), compared to individuals with no CVD-RFs, whereas 7–28% of RA patients would be classified as high-risk individuals (absolute 10-year risk of fatal CVD ≥ 5%) at baseline, depending on which SCORE algorithm was used and if the EULAR 1.5 multiplication factor was applied or not.

**Table 2:** Estimated risk age, relative risk, and absolute risk

**Risk age**	**All patients**
Cardiovascular risk age in years, median (IQR)	59 (48, 69)
Vascular age in years, median (IQR)[SCORE-Hrisk algorithm]	57 (46, 67)
Vascular age in years, median (IQR)[SCORE-Lrisk algorithm]	56 (45, 66)
Vascular age in years, median (IQR)[SCORE-HDL-c-Hrisk algorithm]	58 (49, 67)
Vascular age in years, median (IQR)[SCORE-HDL-c-Lrisk algorithm]	56 (47, 65)
**Relative risk**	
Relative risk of 1, *n* (%)	562 (34.4)
Relative risk of 2, *n* (%)	473 (28.9)
Relative risk of 3, *n* (%)	316 (19.3)
Relative risk of 4, *n* (%)	124 (7.6)
Relative risk of 5, *n* (%)	60 (3.7)
Relative risk of 6, *n* (%)	50 (3.1)
Relative risk of 7, *n* (%)	32 (2.0)
Relative risk of 8, *n* (%)	10 (0.6)
Relative risk of 10, *n* (%)	7 (0.4)
Relative risk of 12, *n* (%)	2 (0.1)
**Individuals with an absolute 10-year risk of fatal CVD ≥ 5%**	
SCORE-Hrisk algorithm, *n* (%)	295 (18.0)
SCORE-Lrisk algorithm, *n* (%)	131 (8.0)
SCORE-HDLc-Hrisk algorithm, *n* (%)	305 (18.9)
SCORE-HDLc-Lrisk algorithm, *n* (%)	111 (6.9)
mSCORE-Hrisk algorithm, *n* (%)	447 (27.3)
mSCORE-Lrisk algorithm, *n* (%)	248 (15.2)
mSCORE-HDLc-Hrisk algorithm, *n* (%)	461 (28.2)
mSCORE-HDLc-Lrisk algorithm, *n* (%)	235 (14.4)

*IQR* inter-quartile range, *SCORE* Systematic Coronary Risk Evaluation, *Hrisk* high-risk country, *HDLc* high-density lipoprotein-cholesterol, *Lrisk* low-risk country, *mSCORE* modified SCORE using a 1.5 multiplication factor, *CVD* cardiovascular disease, *BP* blood pressure, *TC* total cholesterol. Estimated risk age according to the cardiovascular risk age and the various vascular age models, relative risk, and absolute risk of cardiovascular disease in RA patients at baseline. Data are also shown separately for patients who did and did not experience CVD events during follow-up

**Fig. 1:**
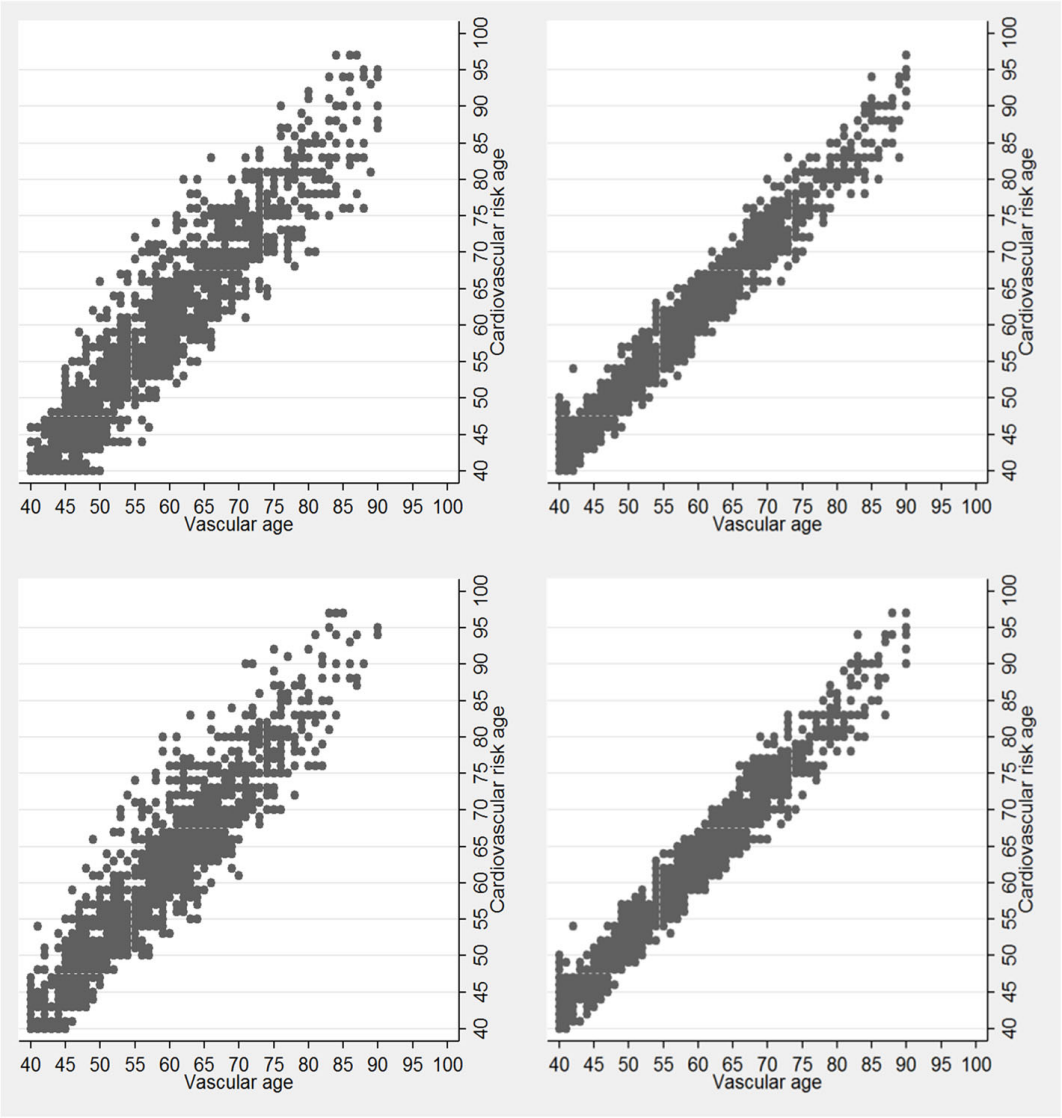
Agreement in estimated risk age by the cardiovascular risk age and vascular age models. Cardiovascular risk age estimations versus cardiovascular risk age calculations using the SCORE algorithms with (left panels) or without HDL-C (right panels) for high-risk countries (top panels) and low-risk countries (bottom panels)

The scatter plot (Fig. 1) gives a graphical presentation of the agreement between estimated risk age according to the cardiovascular risk age model and the various vascular age models. Linear regression analyses, calculating the correlation between the risk age models, yielded *R*^2^ values ranging from 0.87 to 0.97. Moreover, comparison of cardiovascular risk age and various vascular age models revealed that risk age estimations differed ≥ 5 years in 15–32% of observations. The most extreme risk age difference was 21 years in which a female non-smoker, aged 69 years, sBP of 151 mmHg and HDL-c and TC of 2.8 and 7.5 mmol/L, respectively, had a cardiovascular risk age of 80 years, and a vascular age of 59 according to the vascular age model (using the SCORE HDL-c algorithm for low-risk countries).

Overall, the risk age algorithms had c-indices ranging from 0.68 to 0.72, with standard errors (SE) around 0.03 (Table 3). The cardiovascular risk age model had the lowest c-index. Applying the EULAR 1.5 multiplication factor to the estimated risk do not change the risk ranking of subjects and thereby have no impact on concordance. Table 3 also presents the c-indexes when stratifying on sex and RA disease related characteristics. Certain characteristics were associated with low concordance, but standard errors were high. Additional analyses restricted to only European cohorts and/or including patients on LLT and/or AntiHT gave comparable results (data not shown). Finally, we evaluated the concordance of risk models for each center, which revealed wide ranging c-indexes (data not shown). For centers in which CVD events did not occur during the observation period, concordance could not be calculated (data not shown).

**Table 3:** Discriminatory ability according to stratification by RA disease characteristics

		**Sex**			**RF**		**ACPA**		**ACPA/RF**		**bDMARDs**	
	**All**	**Male**	**Female**		**Positive**	**Negative**	**Positive**	**Negative**	**Positive**	**Negative**	**Use**	**No use**
**Cardiovascular risk age**	0.68 (0.03)	0.7 (0.05)	0.63 (0.05)		0.68 (0.04)	0.68 (0.08)	0.72 (0.04)	0.61 (0.06)	0.71 (0.04)	0.65 (0.1)	0.78 (0.14)	0.67 (0.03)
**Vascular age** [H-risk]	0.71 (0.03)	0.72 (0.05)	0.67 (0.04)		0.7 (0.04)	0.77 (0.07)	0.74 (0.04)	0.67 (0.06)	0.74 (0.04)	0.75 (0.08)	0.78 (0.14)	0.71 (0.03)
**Vascular age** [L-risk]	0.71 (0.03)	0.72 (0.05)	0.67 (0.04)		0.7 (0.04)	0.76 (0.07)	0.74 (0.04)	0.67 (0.06)	0.73 (0.04)	0.74 (0.08)	0.8(0.14)	0.71 (0.03)
**Vascular age** [HDLc H-risk	0.71 (0.03)	0.71 (0.05)	0.68 (0.04)		0.7 (0.03)	0.78 (0.07)	0.73 (0.04)	0.69 (0.06)	0.72 (0.04)	0.77 (0.08)	0.78 (0.14)	0.71 (0.03)
**Vascular age** [HDLc L-risk]	0.72 (0.03)	0.72 (0.05)	0.68 (0.04)		0.7 (0.03)	0.76 (0.07)	0.74 (0.04)	0.69 (0.06)	0.73 (0.04)	0.75 (0.08)	0.79 (0.14)	0.71 (0.03)
	**Glucocorticoids**		**DAS28**		**RA disease duration**			**Methotrexate**			**sDMARDs**	
	**Use**	**No use**	**< 3.2**	**≥ 3.2**	**≤ 1 year**	**> 1 year**		**Use**	**No use**		**Use**	**No use**
**Cardiovascular risk age**	0.57 (0.08)	0.71 (0.04)	0.6 (0.09)	0.69 (0.04)	0.69 (0.03)	0.6 (0.08)		0.67 (0.09)	0.68 (0.03)		0.62 (0.08)	0.69 (0.03)
**Vascular age** [H-risk]	0.61 (0.08)	0.74 (0.03)	0.67 (0.09)	0.72 (0.03)	0.73 (0.03)	0.63 (0.08)		0.68 (0.09)	0.72 (0.03)		0.64 (0.08)	0.73 (0.03)
**Vascular age** [L-risk]	0.59 (0.08)	0.74 (0.03)	0.67 (0.09)	0.71 (0.03)	0.73 (0.03)	0.63 (0.08)		0.7 (0.09)	0.71 (0.03)		0.65 (0.08)	0.72 (0.03)
**Vascular age** [HDLc H-risk	0.6 (0.08)	0.74 (0.03)	0.66 (0.09)	0.72 (0.03)	0.73 (0.03)	0.63 (0.08)		0.69 (0.09)	0.72 (0.03)		0.65 (0.08)	0.72 (0.03)
**Vascular age** [HDLc Lrisk]	0.62 (0.08)	0.74 (0.03)	0.67 (0.09)	0.72 (0.03)	0.73 (0.03)	0.64 (0.08)		0.71 (0.09)	0.72 (0.03)		0.66 (0.08)	0.72 (0.03)

Concordance index (standard error) presenting the discriminative ability in ranking individuals who do or do not experience cardiovascular events correctly as high and low risk. Values are presented for various risk age models, including vascular age estimations derived from application of different Systematic Coronary Risk Evaluation algorithms with or without use of high-density lipoprotein-cholesterol (HDLc) for high- and low-risk countries (H-risk and L-risk). Individuals were also stratified according to sex, rheumatoid factor (RF), and anti-cyclic citrullinated peptide antibody (ACPA) positivity, use of biologic or synthetic disease modifying antirheumatic drugs (bDMARDs and sDMARDs), disease activity score using 28-joint count (DAS28), and duration of rheumatoid arthritis (RA)

Significance and innovations

• Risk age estimations have been advocated in current guidelines for CVD prevention

• The two proposed risk age models have not been validated

• Our results indicate comparable performance of risk age models in rheumatoid arthritis patients

## Discussion

Using longitudinal data with CVD outcomes from rheumatic outpatient clinics from the total ATACC-RA cohort, we have revealed comparable ability of various risk age models to rank RA patients in terms of time to CVD events. Interestingly, the cardiovascular risk age chart, which is based on quite wide CVD-RF intervals (e.g., sBP 120/140/160/180 mmHg), had only a slightly lower c-index (0.68) than the other risk age models, indicating comparable performance in correct ranking of individuals in terms of future CVD risk. Thus, although risk age estimations frequently differ 5 years or more [15], their discriminative ability is very similar.

Among the included RA patients, the concordance was about 0.7 for all risk age models and similar to the SCORE algorithms they are based on, which had a c-index of 0.71–0.72. Thus, c-indices were somewhat lower than what was reported for the general European population in the original SCORE paper. Conroy et al. found c-indices of 0.81 and 0.74 for high- and low-risk countries, respectively [8].

Evaluating risk age models by investigating time to event and using a composite of CVD events, the cardiovascular risk age model and the vascular age models all had comparable c-statistics around 0.7. In comparison, a concordance of 0.5 implies a discriminative ability no better than pure chance, whereas c-statistics approaching 0.60 to 0.75 are sometimes expressed as demonstration of “possibly helpful discrimination” and > 0.75 as “clearly useful discrimination” (although this is a criticized practice) [20].

External validation of the SCORE algorithm, from which the cardiovascular risk age and vascular age models are based on and calculated from, respectively, have revealed wide ranging c-statistics. In a review by Damen et al., reported c-indices ranged from 0.62 to 0.91 in different European and non-European study populations [6]. Comparisons of c-indices across populations are hampered due to factors such as differences in age distributions. However, for analyses in these cohorts, several additional explanations of the observed suboptimal concordance are plausible. The SCORE algorithm was developed for the European population. In our analyses, European and non-European cohorts were included. Additional analyses restricted to data from European cohorts were performed, but comparable c-indices were found. We also performed CVD risk estimations using both algorithms for high- and low-risk countries. In the main analyses, we pooled data across centers to increase the numbers and observation time (total person years at risk), but a limitation is the heterogeneity between the various cohorts. There was wide range of the c-indexes and standard errors across each unique center included in the analyses.

In RA, inflammatory disease activity, disease duration, and usage of GCs, sDMARDs, and bDMARDs are all factors that may influence overall risk of CVD [21–28]. In a recent study, Crowson et al. demonstrated that albeit conventional CVD-RFs accounted for half (49%) of CVD events in RA, high-grade inflammation and RA characteristics explained about 30% of the CVD risk [29]. However, the prediction models we evaluated only assess CVD risk related to conventional CVD-RFs. Furthermore, RA patients without known CVD have high occurrence of atherosclerotic plaques even in the case of only moderate estimated absolute risk, justifying the use of carotid ultrasound as a supplement in CVD risk stratification [30].

The latest EULAR recommendations on CVD risk management underline that rheumatic disease activity should be controlled to lower overall risk of CVD [11]. RA-related characteristics may also complicate the interpretation of conventional CVD-RFs. The lipid paradox denotes the phenomena in which low lipid levels due to elevated inflammation is associated with an increased risk of CVD [31]. Thus, a future RA-specific CVD risk algorithm should possibly weight lipid levels according to the disease activity. Regarding CVD prediction models, if important CVD-RFs are left out or not weighted appropriately, then concordance will be impaired.

In this paper, we aimed to evaluate the influence of RA disease characteristics on the performance of risk age models in ranking individuals correctly as high(er)- or low(er)-risk individuals. However, our findings were inconclusive due to the lack of statistical power resulting from the small number of participants included and/or short observation time with few events occurring. Underreporting of CVD events during the follow-up time is also possible, especially since RA patients may suffer from asymptomatic CVD events [32]. The inconclusive results on the association of RA disease characteristics and the c-index values due to large standard errors could have been different with a longer follow-up time and more participants. In time-to-event analyses, consideration of informative and interval censoring is also required. Only data on disease activity and sDMARD and bDMARD treatment were available at baseline. Surprisingly, a high rate of RA patients was not using sDMARDs and bDMARDs at study inclusion. Although this should be considered before extrapolation of our results to other RA cohorts, this may be partly explained by that a high rate of RA patients included in these analyses had short disease duration (explaining why some were methotrexate naive) and also to differences across different nations (explaining why some were bDMARDs naïve). Another limitation to this multi-center study is the lack to control that BP measurements were conducted similarly. Data on family history of premature CVD were also lacking. Among eligible patients, estimation of risk age were not possible in 338–357 individuals when using prediction models without or with HDL-c, respectively, due to missing data on sBP (*n* = 114), TC (*n* = 205), HDL-c (*n* = 219), and current smoking (*n* = 119).

There are also limitations with c-index calculation since it reports concordance based on ranks and not on the magnitude of risk differences. Consequently, in the case of very similar and only slightly different risk ages across subjects, the CVD prediction model’s discriminatory ability will be impaired. Moreover, concordance only describes one feature regarding the predictive ability of a risk model. Calibration, a comparison of the number of expected events to the number of observed events, is another important property regarding validation of prediction models [33]. However, in contrast to models predicting absolute risk, calibration cannot be performed in prediction models using the risk age concept.

The risk age models we have validated are derived from the SCORE algorithms which calculate absolute risk of fatal CVD. In the original SCORE publication, the authors argued that developing a CVD prediction model based on non-fatal CVD events are prone to errors due to misclassification. Non-fatal events are also of clinical importance, and it has been suggested to convert SCORE with a multiplier to estimate fatal and non-fatal events [7, 34]. However, Jørstad et al. found that the ratio of risk of fatal to fatal plus non-fatal CVD was largely dependent on age and sex and, consequently, a fixed multiplication factor was not applicable [35]. Since risk age communicates the detrimental effects of modifiable CVD-RFs on overall CVD risk and/or life expectancy, it is an attractive concept, also informing patients on the benefit of optimizing CVD-RFs. In the review by Groenewegen et al., it is argued that the perspicuous risk age concept might improve communication about CVD risk and possibly patient adherence to CVD preventive strategies (e.g. lifestyle changes and/or cardio-protective medication) [12]. CVD risk assessment is especially important in RA due to the high prevalence of modifiable CVD-RFs [36–38]. However, whether the risk age is an intuitive concept and if it has incremental value beyond absolute and relative risk calculation needs further evaluation.

To our knowledge, this is the first study comparing the discriminative ability of the risk age models proposed for use as supplements to CVD risk evaluation in the ESC guidelines for the general population [7, 12]. Not surprisingly, our study supports the notion that risk age models perform similarly to the SCORE algorithms in ranking individuals correctly as high or low risk of CVD events. Despite that risk age estimations frequently differ 5 years or more, the current risk age models based on SCORE perform almost equivalently in terms of concordance.

## Conclusions

Albeit different CVD risk age algorithms yield marked disparities in a substantial proportion of patients, the cardiovascular risk age and vascular age models have comparable performance in predicting CVD events in RA patients. Furthermore, their concordance is equivalent to the SCORE algorithms they are derived from. The influence of RA disease characteristics on the predictive ability of these prediction models remains inconclusive. Further evaluation is also required to assess whether use of risk age estimations in clinical practice is beneficial for CVD-RF reduction and, ultimately, prevention of CVD events.

## Data Availability

The dataset used and/or analyzed during the current study are available from the corresponding author on reasonable request.

## Abbreviations

ACPA: Anti-citrullinated protein antibodies
AntiHT: Antihypertensive treatment
ATACC-RA: A Trans-Atlantic Cardiovascular Consortium for Rheumatoid Arthritis
bDMARDs: Biologic disease-modifying anti-rheumatic drugs
BMI: Body mass index
CVD: Cardiovascular disease
CVD-RFs: CVD risk factors
c-statistics: Concordance statistics
EULAR: European League Against Rheumatism
ESC: European Society of Cardiology
GC: Glucocorticoids
HDL-c: High-density lipoprotein cholesterol
LLT: Lipid-lowering therapy
LDL-c: Low-density lipoprotein cholesterol
RA: Rheumatoid arthritis
RF: Rheumatoid factor
SE: Standard errors
sDMARDs: Synthetic disease-modifying anti-rheumatic drugs
SCORE: Systematic Coronary Risk Evaluation
sBP: Systolic blood pressure
TC: Total cholesterol
